# CMTM6 and CMTM7: New leads for PD‐L1 regulation in breast cancer cells undergoing EMT

**DOI:** 10.1002/jcb.30273

**Published:** 2022-05-16

**Authors:** Malina Xiao, Caroline Duhem, Anwar Chammout, Guy Berchem, Bassam Janji

**Affiliations:** ^1^ Tumor Immunotherapy and Microenvironment Group, Department of Cancer Research Luxembourg Institute of Health Luxembourg City Luxembourg; ^2^ Department of Hemato‐Oncology Centre Hospitalier du Luxembourg Luxembourg City Luxembourg; ^3^ Department of Oncology, Faculty of Medicine University of Aleppo Aleppo Syria

**Keywords:** breast cancer, CMTM6, epithelial‐to‐mesenchymal transition, immuno‐oncology, immunotherapy, PD‐1, programmed death‐ligand 1 (PD‐L1)

## Abstract

Programmed death‐ligand 1 (PD‐L1) expression has long been used as a biomarker to stratify patients with cancer who will benefit from anti‐PD‐1/PD‐L1 immunotherapy. However, the use of PD‐L1 as a biomarker to guide treatment decisions has recently been called into question due to its dynamic and heterogeneous expression within each tumor and among different tumors as well as during tumor cell plasticity. Therefore, understanding the molecular basis of PD‐L1 expression would enable delineating its value as a reliable biomarker in the clinic. Here, we provide our perspective on the involvement of CMTM6 and CMTM7 as new lead candidates for the regulation of PD‐L1 in breast tumors undergoing an epithelial to mesenchymal transition.

Over the past decades, immunotherapy based on blocking the immune checkpoints has emerged as one of the most promising therapies to fight cancers and is in the process of fundamentally reshaping the treatment of cancer.^1^

Cytotoxic T lymphocyte‐associated molecule‐4 (CTLA‐4), programmed cell death receptor‐1 (PD‐1), and programmed cell death receptor‐1 ligand (PD‐L1) are widely used in the clinic and the best described immune checkpoint blockades (ICBs) so far. However, clinical data highlight that the objective response rate to anti‐PD‐1/PD‐L1 antibodies varies across tumor types (reviewed in ref. [2] and summarized in Table 1).

**Table 1:** Objective response rate (ORR) to anti‐PD‐1/PD‐L1 antibodies in different cancer types of male and female is reported as a percentage

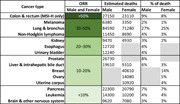

*Note*: The estimated deaths and the % of death in male and female are reported from the American Cancer Society, Cancer Facts and Figures 2017—https://www.cancer.org/research/cancer-facts-statistics/. Table adapted from ref. [2].

The clinical development of an antibody blocking the immune checkpoint PD‐1 is still at the epicenter of the immuno‐oncology landscape. However, this enthusiastic vision has been challenged by the clinical observation that only few patients benefit from the remarkable clinical remissions achieved by ICBs.^3^

To broaden the benefit of ICBs, notably anti‐CTLA‐4 and anti‐PD‐1/PD‐L1, immuno‐oncology research is now pushing toward combining several ICBs. Despite the substantial number of clinical trials assessing combinatorial ICB approaches with anti‐PD‐1/PD‐L1, the therapeutic benefit of a considerable number of them was disappointing and did not meet clinical expectations (minimal survival benefit and high toxicity).^4^

Several mechanisms have been described to affect the efficacy of ICBs. Briefly, tumors displaying high neoantigen load and increased number of DNA mutations, due to a defect in DNA repair mechanism, are more prone to respond to ICB than those having low number of mutations and decreased neoantigen load.^5^ Furthermore, the immune landscape status of the tumor microenvironment is also a major factor predicting the response to ICBs. Indeed, the density and location of immune cells in the tumor microenvironment have led to establishing the concept of “cold” immune desert tumors, which are not eligible and not responding to ICBs, and “hot” inflamed immune infiltrated PD‐L1 expressed tumors, which are eligible and responding to ICBs.^6^

Regardless of the mechanism involved in affecting the response to ICBs, we believe that furthering our understanding of the molecular mechanisms involved in the regulation of PD‐1 and its ligand PD‐L1 can contribute to the design of more effective anti‐PD‐1/PD‐L1‐based therapies and bring substantial benefits to cancer patients.

PD‐1 is a cell surface immune checkpoint receptor expressed on T cells. Its interaction with PD‐L1 or PD‐L2, expressed on tumor cells, activates downstream signaling pathways and inhibits T cell activation. Several tumor cells express high levels of PD‐L1 and exploit PD‐L1/PD‐1 signaling to induce T cell immune suppression.^7^ Similar to their physiological function of helping normal cells to maintain self‐tolerance, highly aggressive cancer cells can hijack the immune cell attack by overexpressing PD‐L1, which allows the tumor to escape from immune surveillance.^8^, ^9^

Among the most described processes involved in the acquisition by tumor cells of highly aggressive and metastatic properties is the epithelial‐to‐mesenchymal transition (EMT). EMT is a physiological process, initially described during embryogenesis, whereby a polarized epithelial cell loses its basal‐apical polarity to acquire dynamic and invasive properties typical of a mesenchymal state. In line with the high plasticity of tumor cells, the EMT process can be reverted using a mechanism referred to as mesenchymal‐to‐epithelial transition (MET).^10^ EMT is coordinated by series of master EMT‐inducing transcription factors (EMT‐TFs) including (i) zinc‐finger binding transcription factors SNAI1 (SNAIL) and SNAI2 (SLUG); and (ii) zinc finger E‐box‐binding homeobox 1/2 (ZEB1/2), TWIST, and lymphoid enhancer‐binding factor‐1 (LEF‐1). EMT‐TFs bind to the promoter region of several genes to regulate their expression.^11^ Mounting experimental evidence supports that EMT‐TF promotes drug resistance, stemness, immune evasion, and immune suppression.^12^ According to the epithelial or mesenchymal status of cells or tumors, an EMT scoring system has been previously established based on the expression of different epithelial and mesenchymal markers. Tumors or cells with a negative EMT score are considered highly epithelial, while those with a positive EMT score are considered highly mesenchymal.^13^

Several lines of evidence highlight the critical role of EMT‐related features (such as loss of E‐cadherin) in tumor escape from immune surveillance (reviewed in ref. [14]). Briefly, the induction of EMT by hypoxia upregulates CCL20 in hepatocellular carcinoma cell lines. Such upregulation induces the expression of indoleamine 2, 3‐dioxygenase (IDO) in macrophages and increases the numbers of Foxp3+ regulatory T cells (Treg cells) and subsequent decreased T‐cell proliferation^15^ (Figure 1A). In vitro coculture of T, B, or NK cells with cancer cell lines undergoing EMT decreased lymphocyte proliferation and increased NK and T‐cell apoptosis through a mechanism involving IDO.^16^

**Figure 1:**
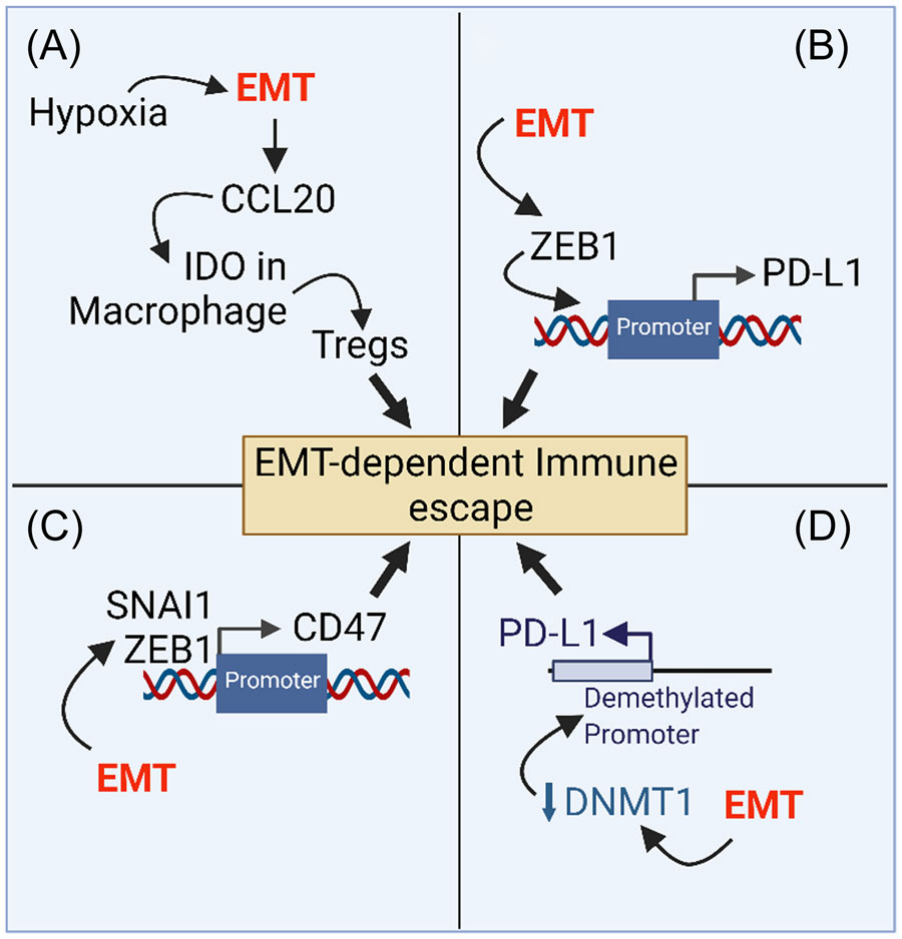
Major EMT‐dependent mechanisms involved in the regulation of immune checkpoints and tumor immune escape. (A) Driving EMT by hypoxia in tumor cells upregulates the expression of CCL20 and Such upregulation increases IDO in macrophages. IDO induces a metabolic switch in macrophages and increases Foxp3+ Treg cells. (B) In mesenchymal breast cancer cells, ZEB‐1 strongly induces PD‐L1 expression. (C) The EMT‐TFs SNAI1 and ZEB1 upregulate CD47 by direct binding of these EMT‐TFs to the E‐boxes in the human CD47 promoter. (D) Driving EMT by TGF‐β decreases the level of DNA‐methyltransferase 1 (DNMT1), resulting in PD‐L1 promoter demethylation. Driving EMT by TNF‐α induces NF‐κB pathway and promotes the expression of demethylated PD‐L1 promoter. EMT, epithelial‐to‐mesenchymal transition; IDO, indoleamine 2, 3‐dioxygenase; TF, transcription factor.

We have previously reported that PD‐L1 is differentially upregulated in MCF‐7 human breast cancer cell sub‐clones undergoing EMT and resulted in tumor cell resistance to CTL‐mediated killing. Mechanistically, we revealed that silencing the EMT‐TF ZEB‐1 and overexpressing miR200 family members, reported to suppress EMT in EMT‐activated mesenchymal cells, strongly decreased PD‐L1 expression. Interestingly, siRNA targeting of PD‐L1 or PD‐L1 blocking antibody restored the susceptibility of highly resistant EMT‐activated MCF‐7 sub‐clones to CTL‐mediated killing^14^, ^17^, ^18^ (Figure 1B).

The role of EMT‐TF is not restricted to PD‐L1. Accumulating new data revealed that EMT regulates the macrophage immune checkpoint CD47, a transmembrane immune checkpoint protein receptor expressed on the surface of tumor cells. CD47 delivers a strong “don't eat me signal” upon binding to its ligands signal regulatory protein α (SIRPα) on the surface of macrophages and dendritic cells, resulting in phagocytosis blockade.^19^ We provided evidence that CD47 is upregulated in different EMT‐activated human breast cancer cells. Mechanistically, we revealed that SNAI1 and ZEB1‐dependent upregulation of CD47 occurs by direct binding of these EMT‐TFs to the E‐boxes in the human CD47 promoter. Furthermore, in silico analysis of the Cancer Genome Atlas (TCGA) and METABRIC data sets from thousands of patients with breast cancer revealed that CD47 expression positively correlates with SNAI1 and Vimentin. At the functional level, we showed that macrophages less efficiently phagocytosed EMT‐activated clones derived from MCF‐7 cells compared with the parental epithelial MCF‐7 cells. The phagocytosis of EMT‐activated clones was rescued by a CD47 blocking antibody or by genetically targeting SNAI1, ZEB1, or CD47^20^ (Figure 1C).

Based on the data described above, it is tempting to speculate that combining anti‐CD47 and anti‐PD‐L1 agents would simultaneously reactivate both innate (macrophage checkpoint CD47) and adaptive immunity (T‐lymphocyte checkpoint PD‐1). Such reactivation leads to durable long‐lasting antitumor immune responses in highly aggressive and metastatic breast cancers undergoing EMT.

In addition to the transcriptional regulation, evidence indicates that PD‐L1 can also be regulated by epigenetic modification during the EMT process. Indeed, driving EMT by TGF‐β decreased the level of DNA‐methyltransferase 1 (DNMT1), resulting in PD‐L1 promoter demethylation. However, driving EMT by TNF‐α induced NF‐κB pathway and promoted the expression of demethylated PD‐L1 promoter in NSCLC^21^ (Figure 1D).

The analysis of cancer patient data revealed that high EMT score is associated with high expression of PD‐1, PD‐L1, CTLA4, OX40L, and PD‐L2 in several cancer types.^22^

In lung adenocarcinoma, the expression of PD‐L1, PD‐L2, PD‐1, TIM‐3, B7‐H3, BTLA, and CTLA‐4 is positively correlated with EMT.^23^ Similarly, a positive correlation between PD‐L1 expression and EMT phenotype has been reported in high‐risk hepatocellular carcinoma patients.^24^ In Lung cancer cell lines, a therapy inducing E‐cadherin downregulation results in PD‐L1 downregulation.^25^ Based on the data described above, we believe that a relationship between the objective response rate (ORR) to anti‐PD‐1/PD‐L1 and the EMT score would exist for some tumor types. Indeed, Table 2 showed that colorectal tumors displaying epithelial EMT score demonstrated >50% ORR to anti‐PD‐1/PD‐L1 therapy. However, brain tumors having mesenchymal EMT score showed only <10% ORR to anti‐PD‐1/PD‐L1 therapy.

**Table 2:** Objective response rate (ORR) to anti‐PD‐1/PD‐L1 antibodies and the EMT score of different cancer types

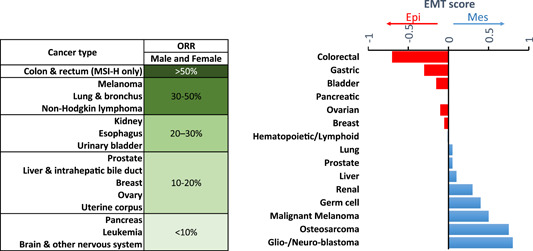

*Note*: The EMT score was plotted for each tumor type based on data reported in ref. [13]. Tumors with a negative EMT score are considered highly epithelial (in red), while those with a positive EMT score are considered highly mesenchymal (in blue).

Abbreviation: EMT, epithelial‐to‐mesenchymal transition.

Chemokine‐like factor‐like (CKLF‐like) proteins containing MARVEL transmembrane domain (CMTM) are proteins involved in the regulation of transmembrane and secretory proteins trafficking.^26^ The CMTM family contains eight members (CMTM1–8) located in three distinct genes. CMTM1–4 members are located on chromosome 16q, CMTM5 is found on chromosome 14q11.2, and CMTM6‐8 members are located on chromosome 3p22.3.^27^ CMTM proteins are mostly found on the cell surface and cytoplasm. They are upregulated in several tumors and reported as potential tumor suppressor genes associated with development and metastasis.^28^ By using a genome‐wide CRISPR–Cas9 screen, CMTM6 has been identified as a major regulator of the cell surface expression of PD‐L1 in various cancers, including breast. Although not required for PD‐L1 maturation, CMTM6 co‐localizes and binds PD‐L1 to maintain its expression on the cell surface. Targeting CMTM6 significantly reduced the cell surface expression of PD‐L1 even in the presence of IFNγ stimulation, highlighting the major interest of developing CMTM6 inhibitors to limit the IFNγ‐dependent induction of PD‐L1 expression in vivo. In the recycling endosomes, CMTM6 prevents PD‐L1 from being targeted for lysosome‐mediated degradation. The functional role of CMTM6 was demonstrated by showing that the decrease in PD‐L1 expression following CMTM6 depletion significantly enhances T‐cell mediated tumor cell killing in vitro and in vivo.^29^ Interestingly, CMTM4 plays a role as a backup regulator of PD‐L1 expression only in the absence of CMTM6.^29^, ^30^ Although the key role of CMTM6 on the expression and stabilization of PD‐L1 is now well established, the mechanism regulating the expression of CMTM6 remains undefined.^26^, ^27^

Based on data showing that EMT‐TFs regulate the expression of PD‐L1, it is tempting to speculate that EMT‐dependent overexpression of PD‐L1 occurs through upregulating CMTM6 expression. If so, it is crucial to determine (i) whether CMTM6 is the only protein of the CMTM family to be regulated by EMT; (ii) whether CMTM6 operates alone or in coordination with other CMTM members; and (iii) what is the relative fraction of PD‐L1 on the cell surface to be regulated by CMTM6 in the context of EMT?

To address these issues, we first investigated whether CMTM6 mRNA correlates with EMT score in breast cancer cells and with VIMENTIN mRNA expression in triple‐negative breast cancer patients (TNBC).^31^ In silico analysis performed on several breast cancer cell lines described in the Cancer Cell Line Encyclopedia (CCLE) database showed a positive correlation of CMTM6 mRNA expression with the EMT score. Such results have been validated in METABRIC TNBC patients' cohort reported in the TCGA database by showing that CMTM6 mRNA expression positively correlated with VIMENTIN mRNA. Our in silico data have been experimentally validated using an in vitro cell model consisting of epithelial MCF‐7 and mesenchymal MDA‐MB‐231 breast cancer cells. Indeed, we showed that both PD‐L1 and CMTM6 were upregulated at both the mRNA and protein levels in MDA‐MB‐231 cells relative to MCF‐7 cells. We provided evidence that the upregulated expression of PD‐L1 in MDA‐MB‐231 was dependent on CMTM6 because the cell surface expression of PD‐L1 was significantly decreased upon silencing CMTM6. To further confirm the role of EMT in the regulation of CMTM6, we used a genetic approach to induce a mesenchymal switch in MCF‐7 epithelial cells. The MCF‐7 cells were stably transduced with a doxycycline‐inducible SNAI1 vector (MCF‐7 iSNAI1). We observed that the doxycycline‐treated cells (hereafter referred to as MCF‐7^Mes^) completely shifted into a mesenchymal phenotype compared with the untreated cells (hereafter referred to as MCF‐7^Epi^). This mesenchymal shift was associated with the overexpression of SNAI1 together with the decrease of the epithelial marker E‐cadherin. Strikingly, PD‐L1 and CMTM6 protein levels were both significantly upregulated in MCF‐7^Mes^ relative to MCF‐7^Epi^ cells. To determine whether the overexpression of PD‐L1 was dependent on CMTM6 upregulation in MCF‐7^Mes^ cells, we depleted CMTM6 by siRNA. Surprisingly, we observed a slight but significant decrease (estimated at 25%) in total PD‐L1 expression following CMTM6 silencing in MCF‐7^Mes^. Our results incited us to investigate whether other members of the CMTM cooperate with CMTM6 in the regulation of PD‐L1 in cells undergoing EMT. We first evaluated in silico which CMTM members were regulated during EMT. Our data showed that CMTM3 and CMTM7 mRNA were upregulated in EMT‐activated breast cancer cells. Using genetic approaches, we provided evidence that although both CMTM3 and CMTM7 are upregulated in MCF‐7^Mes^, only CMTM7 cooperates with CMTM6 to regulate together a substantial part of total PD‐L1 (estimated at 50%).^31^

Taken together, our findings have begun to unravel the mechanism underlying the regulation of CMTM6, a major protein involved in PD‐L1 stabilization (Figure 2). We demonstrated that a considerable part of PD‐L1 that is overexpressed during the EMT process is regulated via CMTM6 and CMTM7 in breast cancer cells. Considering that SNAI1 can induce ZEB1 expression and that both SNAI1 and ZEB1 share common E‐boxes binding sites,^32^, ^33^ it would be important to determine whether ZEB1 also contributes to the regulation of CMTM6 and CMTM7 mRNA. Moreover, we cannot rule out that SNAI1, via ZEB1, can directly contribute to the upregulation of PD‐L1 in a CMTM6 and CMTM7‐independent manner. Nevertheless, chromatin immunoprecipitation experiments are warranted to demonstrate the direct binding of SNAI1 to the potential E‐Box motifs in the promoter of CMTM6 and CMTM7. The functional impact of such binding in the transactivation of the genes should also be supported by luciferase reporter assays.

**Figure 2:**
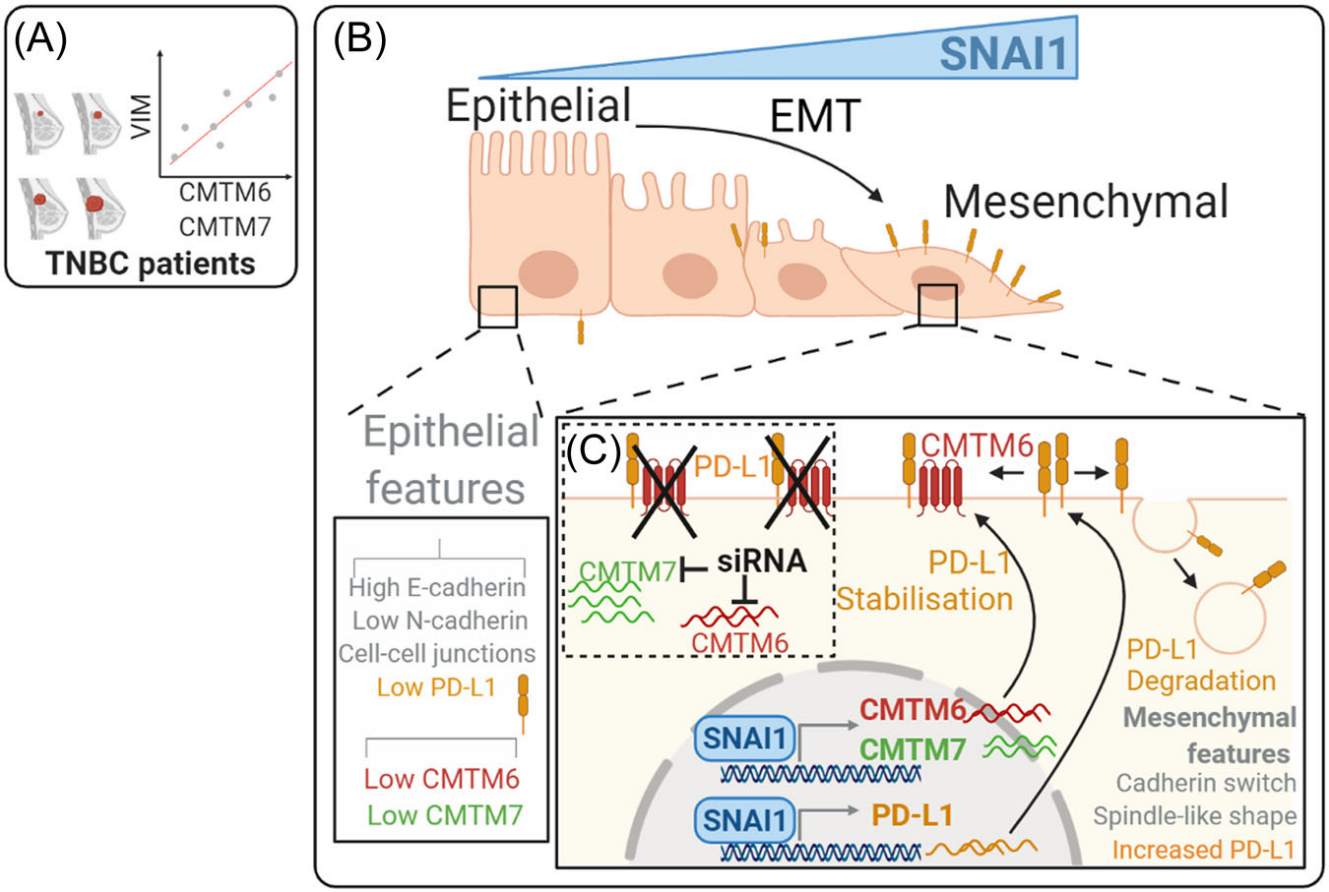
Schematic representation of the involvement of CMTM6 and CMTM7 in the regulation of PD‐L1 in breast tumor cells. (A) In triple‐negative breast cancer (TNBC) patients, in silico data showed a positive correlation between the expression of CMTM6/7 and the EMT marker Vimentin (VIM). (B) Epithelial tumor cells are characterized by high expression of E‐cadherin and low expression of N‐cadherin, PD‐L1, CMTM6, and CMTM7. During EMT, mesenchymal cells express high level of SNAI1, which is associated with a decrease in E‐cadherin, and an increase in PD‐L1 expression. SNAI1 is involved in the transactivation of CMTM6/7, which is involved in the stabilization of PD‐L1 on the cell surface. In mesenchymal cells, SNAI1 is directly involved in the expression of PD‐L1. However, in the absence of CMTM6, PD‐L1 is internalized via the endosome pathways and degraded. (C) Simultaneous targeting of CMTM6 and CMTM7 is sufficient to significantly decrease the expression of PD‐L1 on the surface of mesenchymal tumor cells. EMT, epithelial‐to‐mesenchymal transition.

Finally, the effect of targeting CMTM6 and/or CMTM7 in breast cancer cells undergoing EMT on tumor development, metastasis, and T cell activity should be addressed. In this context, targeting CMTM6 in head and neck squamous cell carcinoma inhibits the growth of squamous carcinoma cell line SCC7, increases CD8^+^ and CD4^+^ T‐cell infiltration, and decreases the proportion of PD‐1^+^, TIM‐3^+^, VISTA^+^, LAG‐3^+^, and B7‐H3^+^ exhausted T cells.^34^

Despite the unprecedented efficacy of the PD‐L1/PD‐1 blockade in cancer immunotherapies, only a small proportion of patients with PD‐L1‐positive tumors achieve a high objective response rate, while others seem to develop resistance to such therapy. We strongly believe that deciphering the molecular mechanisms underlying the regulation of PD‐L1 might inform and direct further preclinical research toward designing an effective ICB‐based cancer immunotherapy. Current evidence indicates that PD‐L1 is regulated by non‐mutually exclusive mechanisms including inflammatory and oncogenic signaling pathways, microRNA, genetic alteration, and posttranslational modifications via CMTM4 and CMTM6.^37^ Therefore, our study provides new mechanistic insight into how tumor cell plasticity regulates the expression of PD‐L1 by operating on two members of the CMTM family. Furthermore, we strongly believe that developing innovative therapies modulating the expression of CMTM family would have a significant clinical relevance notably on circulating tumor cells (CTCs) which are major players in tumor recurrence and metastasis. Indeed, the survival of CTCs relies on genetic aberrations, acquisition of cancer stem cell properties and EMT. It is now well established that CTCs underwent EMT are able to escape immune effector cell attack and resist to chemotherapy. The phenotypic characterization of CTCs revealed that the expression of EMT markers is upregulated in specific tumor types.^38^ Moreover, in 68% of HR^+^ HER‐2^−^ breast cancer patients, isolated CTCs displayed high expression level of PD‐L1.^39^ Therefore, CTCs^PD‐L1‐high^ can escape immune surveillance by two non‐mutually exclusive mechanisms: (i) PD‐L1 on CTCs can interact with PD‐1, expressed on the surface of activated T cells and trigger apoptosis, and (ii) PD‐L1 on CTCs can mediate Tregs to play a role in immunosuppression. Therefore, the high expression of PD‐L1 on CTCs might be used as an escape mechanism from immune surveillance.^40^ Based on these data, it would be interesting to analyze the expression of CMTM family members in CTC^PD‐L1‐high^. Nevertheless, we believe that discovering CMTM6 and CMTM7 as important regulators of PD‐L1 will pave the way for new therapeutic opportunities to restore the immune surveillance of CTCs^PD‐L1‐high^ population, which ultimately improve the efficacy of ICBs in highly aggressive and metastatic tumors.

## AUTHOR CONTRIBUTIONS

Malina Xiao, Caroline Duhem, Anwar Chammout, Guy Berchem, and Bassam Janji equally contributed to writing the manuscript.

## CONFLICTS OF INTEREST

The authors declare no conflicts of interest.

## Data Availability

Not applicable for review manuscript.
